# Cynanoside F Controls Skin Inflammation by Suppressing Mitogen-Activated Protein Kinase Activation

**DOI:** 10.3390/antiox11091740

**Published:** 2022-09-01

**Authors:** Mara Melissa Duarte Fleitas, Seon Sook Kim, Nam Kyoung Kim, Su Ryeon Seo

**Affiliations:** 1 Department of Molecular Bioscience, College of Biomedical Science Kangwon National University, Chuncheon 24341, Korea; 2 Institute of Bioscience & Biotechnology, Kangwon National University, Chuncheon 24341, Korea; 3 R&D Center, Medipeau Inc., Chuncheon 24398, Korea

**Keywords:** cynanoside F, atopic dermatitis, skin inflammation

## Abstract

Atopic dermatitis (AD) is a chronic inflammatory skin disease accompanied by severe itching and dry skin. Currently, the incidence of AD due to excessive activation of immune cells by various environmental factors is increasing worldwide, and research on inflammatory response inhibitors with fewer side effects is continuously needed. Cynanoside F (CF) is one of the pregnane-type compounds in the root of *Cynanchum atratum*, an oriental medicinal herb that has been shown to have antioxidant, antitumor, and anti-inflammatory effects. Although CF has been isolated as a component in *Cynanchum atratum*, the scientific role of CF has not yet been explored. In this study, we evaluated the effect of CF on AD and revealed the mechanism using in vitro and in vivo experimental models. CF significantly reduced lipopolysaccharide (LPS)-induced protein expression levels of interleukin-1β (IL-1β), interleukin-6 (IL-6), and cyclooxygenase-2 (COX-2), which are important proinflammatory mediators in the RAW264.7 macrophage cell line. CF did not inhibit the nuclear factor-kappa B (NF-κB) signaling activated by LPS but significantly reduced the phosphorylation of mitogen-activated protein kinases (MAPKs), such as p38 MAPK, JNK, and ERK. CF consistently inhibited the activity of the activator protein-1 (AP-1) transcription factor, a downstream molecule of MAPK signaling. In addition, in an experiment using an oxazolone-induced AD mouse model, the CF-treated group showed a marked decrease in epidermal thickness, the number of infiltrated mast cells, and the amount of histamine. The mRNA levels of IL-1β, interleukin-4 (IL-4), and thymic stromal lymphopoietin (TSLP) were consistently lowered in the group treated with CF. Moreover, the phosphorylation of c-Jun and c-Fos protein levels, which are the AP-1 components, were lowered in the skin tissues of CF-treated mice. These results provide the first evidence that CF has an inhibitory effect on AD and suggest the possibility of CF being developed as a potential therapeutic agent for AD.

## 1. Introduction

Atopic dermatitis (AD) is a chronic inflammatory skin disease that is accompanied by severe itching and dry skin and usually begins in childhood. Approximately 10 to 20% of children have AD, which usually improves or goes away in adults. However, many people continue to develop symptoms into adulthood [1,2]. Although the cause of AD has not yet been clearly identified, it is reported that genetic, environmental, and immunological factors are involved. In particular, the incidence of AD with excessive activation of immune cells is increasing worldwide due to environmental factors [3].

The immune response of AD involves a Th2-mediated hyperimmune response. Antigen-presenting cells (APCs), such as B cells and neutrophils, bind and present allergens to T cells to induce cell-mediated immune responses. These invoked T cells excessively differentiate into Th2 cells and induce the production of IgE and the differentiation of mast cells through cytokines such as interleukin-4 (IL-4), interleukin-5 (IL-5), and interleukin-13 (IL-13) [4]. Mast cells produce sustainable amounts of histamine, which causes itching, a typical symptom of AD [5]. Excessive scratches from chronic inflammation can damage the skin barrier, exposing it to foreign microorganisms such as bacteria. Lipopolysaccharide (LPS) is a major component of the cell wall of Gram-negative bacteria and increases the production of proinflammatory cytokines such as interleukin-1β (IL-1β), interleukin-6 (IL-6), and tumor necrosis factor-α (TNF-α) and has an inducible effect on cyclooxygenase-2 (COX-2) in macrophages [6]. The induction of COX-2 produces large amounts of prostaglandin E2 (PGE2), which acts to increase blood flow and vascular permeability with the characteristics of inflammation, including redness, warmth, swelling, and pain [7].

Activated macrophages in turn induce Toll-like receptor 4 (TLR4)-mediated intracellular signaling cascades, such as nuclear factor-kappa B (NF-κB) and activator protein-1 (AP-1) as well as mitogen-activated protein kinases (MAPKs), signal transducers and activators of transcription (STATs), and phosphoinositide-3 kinase/AKT (PI3K/AKT) [8]. In particular, NF-κB and AP-1 are important transcription factors that regulate the expression of various inflammatory genes. When macrophages are stimulated with LPS, they activate AKT, STATs, and MAPK signaling pathways to trigger the translocation of NF-κB and AP-1 into the nucleus, leading to the transcription of inflammatory mediators [9].

In AD, inflammatory symptoms can be reduced by suppressing excessive inflammatory reactions, and for this purpose, antihistamines, immunosuppressants, and steroids are used. However, some of these may cause serious side effects in the human body, making continued use difficult [10]. In addition, most patients suffering from AD are infants and children. Therefore, there is a constant need for research on new substances derived from natural products that have fewer side effects and can be used safely in children.

Worldwide, multiple groups have investigated the use of traditional medicinal sources to inhibit and suppress inflammatory responses for treating AD [11,12,13]. *Cynanchum atratum* is an oriental medicinal herb widely distributed throughout China, Korea, and Japan. It belongs to the Asclepiadaceae family and has traditionally been used to treat fever, urinary tract infections, edema, and rheumatic arthralgia [14,15]. Pharmacological investigations have reported that *Cynanchum atratum* has antitumor, anti-amnesic, and anti-acetylcholinesterase activities as well as anti-inflammatory activities [15,16,17].

Cynanoside F (CF) is a pregnane-type steroidal glycoside found within the *Cynanchum atratum* [18,19]. However, the role of CF remains undefined. In this study, we evaluated the effect of CF on AD by using in vitro and in vivo experimental models and found that CF ameliorates AD symptoms by inhibiting the MAPK signaling cascade.

## 2. Materials and Methods

### 2.1. Materials

Cynanoside F (CF) was obtained from Xinyang Laiyao Biotechnology Co., Ltd. (CAS: 1800029-50-8, Beijing, China). Lipopolysaccharide (LPS), 4-ethoxymethylene-2-phenyl-2-oxazolin-5-one (oxazolone), 3-(4,5-dimethylthiazol-2-yl)-2,5-diphenyltetrazoliumm bro-mide (MTT), DMSO, and toluidine Blue were purchased from Sigma-Aldrich Chemicals (St. Louis, MO, USA). The anti-phospho-IκB, anti-phospho-p38, anti-phospho-JNK, anti-phospho-ERK, anti-COX-2, anti-phospho-NF-κB, and anti-phospho-c-Jun antibodies were purchased from Cell Signaling Technology (Danvers, MA, USA). The anti-IL-1β antibody was purchased from R&D Systems (Minneapolis, MN, USA). Anti-GAPDH and anti-c-Fos antibodies were obtained from Santa Cruz Biotechnology (Dallas, TX, USA). Hematoxylin & Eosin (H&E) staining agents were purchased from Abcam (Cambridge, UK). The mouse Histamine ELISA Kit was purchased from Fine Biotech (Wuhan, China). All culture reagents were acquired from Thermo Fisher Scientific (Waltham, MA, USA).

### 2.2. Cell Culture

The RAW264.7 murine macrophage cell line was acquired from ATCC (Manassas, VA, USA). Cells were grown at 37 °C in DMEM enriched with 10% fetal bovine serum (FBS), streptomycin sulfate (100 mg/mL), and penicillin (100 U/mL) in a humidified 5% CO_2_ atmosphere.

### 2.3. Cell Viability Assay

Cell viability was evaluated using an MTT assay. In 96-well plates, RAW264.7 macrophages were seeded at a density of 1 × 10^4^ cells per well and kept at 37 °C for 24 h. The cells were exposed to 0.1 and 1 µM concentrations of CF. After 6 h of treatment, the macrophages were incubated at 37 °C with an MTT solution (5 mg/mL) for 1 h under 5% CO_2_. DMSO (100 μL) was added to dissolve the formazan crystals and a Model 680 microplate reader (Bio-Rad, Hercules, CA, USA) was used to measure the absorbances of the solutions at a wavelength of 570 nm. The experiments were performed in triplicate, and the absorbance value was used to calculate cellular viability, which was then compared to the vehicle-treated control group.

### 2.4. Western Blot Analysis

Cells were lysed in a 1% Nonidet P-40 lysis buffer with protease inhibitors (150 mM NaCl, 50 mM Tris-Cl, pH 8.0, 10% glycerol, 1 mM EGTA, 10 mM NaF, 1 mM Na_3_VO_4_, 0.2 mM phenylmethylsulfonyl fluoride (PMSF), 1 μg/mL leupeptin, and 1 μg/mL aprotinin). The total cell lysates were centrifuged, and the supernatant was separated by SDS-PAGE. Proteins were transferred to nitrocellulose (NC) membranes, and the membranes were blocked in a TBST buffer (137 mM NaCl, 0.05% Tween 20, 20 mM Tris-Cl, pH 7.6) with 5% nonfat milk. The membranes were incubated overnight with the appropriate antibodies at 4 °C. The bands were visualized with ECL (Clarity Western ECL, Bio-Rad, Hercules, CA, USA).

### 2.5. Reporter Gene Assay

RAW264.7 cells were cultivated in a 6-well culture dish. When 80% density was achieved, cells were transfected with either 0.5 µg of pNF-κB-luc (firefly reporter) or 0.5 µg of p-AP-1-luc reporter (firefly reporter) together with 0.1 µg of pTK-luc (renilla reporter) using the Lipofectamine 3000 method according to the manufacturer’s instructions (Invitrogen, Carlsbad, CA, USA). Luciferase activity was assessed after 24 h of transfection using the dual-luciferase assay equipment from Promega (Madison, WI, USA). The firefly activity was normalized to the renilla activity.

### 2.6. Real-Time Quantitative PCR (qRT-PCR)

Total RNA was isolated from cells using a TRIzol reagent (Invitrogen, Carlsbad, CA, USA), and subsequent reactions were performed as previously described [20]. The product was amplified with a SYBR Green real-time PCR Master Mix (Toyobo, Osaka, Japan) with the following primers:

Band intensities were quantified using a real-time quantitative PCR instrument, Aria Mx (Agilent, Santa Clara, CA, USA). Relative changes in target gene expression were calculated by the 2^−^^ΔΔCT^ method [21]. All experiments were performed in triplicate and normalized to β-actin.

### 2.7. Oxazolone-Induced Atopic Dermatitis (AD) Mouse Model

Female SKH1 hairless mice were purchased from Orient Bio Inc. (Seongnam, Korea). The animals were kept in controlled conditions at Kangwon National University’s animal facility with a standard diet and water. The Institutional Animal Care and Use Committee (IACUC) gave its approval for all experiments (KW-191107-2, Kangwon National University, Chuncheon, Korea). The mice were sensitized with 80 μL of 1% oxazolone dissolved in a mixture of acetone and olive oil (3:1) applied to the dorsal skin. After one week of sensitization, 80 μL of 0.1% oxazolone and 50 μL of CF (10 μg/mL) or vehicle (DMSO) were repeatedly applied to the dorsal skin of the mice at 2 day intervals from Day 7 to Day 25. On Day 26, mice were sacrificed, and whole blood along with dorsal tissues were collected for further analysis. Each group consisted of 5 mice.

### 2.8. Histological Analysis

H&E staining was performed on 2–3 μm dorsal skin tissue sections that were fixed overnight in 4% formalin and embedded in paraffin. Histopathological changes were examined by light microscopy (Olympus, Tokyo, Japan) and photographed. The number of infiltrated mast cells was counted followed by toluidine blue staining.

### 2.9. ELISA

Blood samples from AD mice were centrifuged for 20 min at 800× *g*. The supernatant (serum) was transferred into a new tube, and the concentration of histamine was detected in accordance with the manufacturer’s protocol (Fine Biotech, Wuhan, China).

### 2.10. Statistical Analysis

All data analysis was conducted with GraphPad Prism software version 5.01 (GraphPad Software, Inc., San Diego, CA, USA), and the results are presented as the means ± SDs. A Student’s t test was used to examine differences between the experimental and control groups. ANOVA was used to assess comparisons across various groups, followed by Bonferroni post hoc testing. A *p* < 0.05 value was considered statistically significant. * *p* < 0.05; ** *p* < 0.01; *** *p* < 0.001.

## 3. Results

### 3.1. CF Has an Inhibitory Effect on Proinflammatory Cytokine Expression in RAW264.7 Macrophages

**Figure 1:**
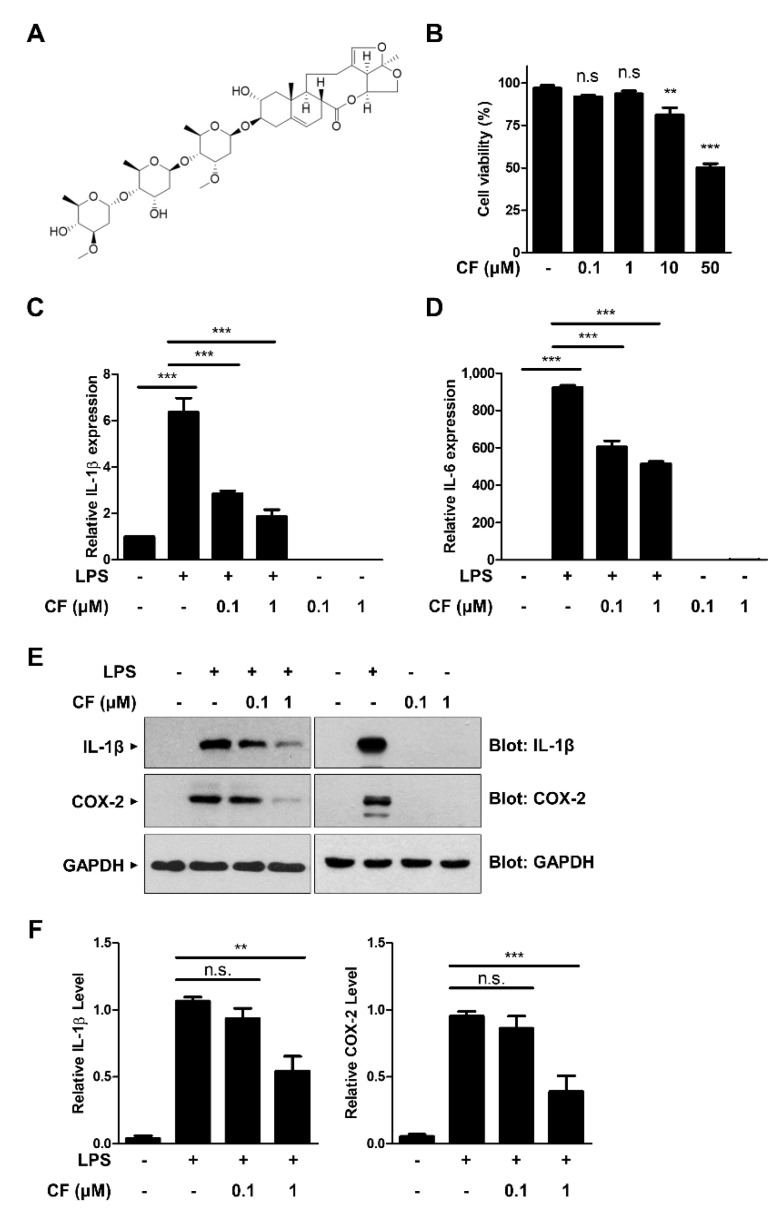
Inhibitory effect of CF on LPS-induced inflammatory signaling. (**A**) The chemical structure of CF. (**B**) RAW264.7 macrophages were treated with the indicated concentrations of CF for 24 h, and the MTT assay was used to determine cell viability. (**C**–**F**) RAW264.7 macrophage cells were pretreated with CF (0.1 and 1 µM) for 30 min before treatment with LPS (500 ng/mL) for 6 h. The mRNA expression levels of IL-1β, IL-6, and β-actin were measured by qRT-PCR with the indicated primer sets (Table 1) and then quantified (**C**,**D**). Cell lysates were immunoblotted with anti-IL-1β and anti-COX-2 antibodies. GAPDH was used as an internal control (**E**,**F**). The graphs are presented as the mean ± SD of three independent experiments. ** *p* < 0.01; *** *p* < 0.001; n.s., nonsignificant.

To evaluate the potential effect of CF on inflammatory signaling, we first determined the noncytotoxic concentration of CF to RAW264.7 murine macrophages. The structure of CF is shown in Figure 1A. RAW264.7 cells were treated with increasing concentrations of CF for 24 h, and the cell viability was evaluated with the MTT assay (Figure 1B). CF did not significantly affect the viability of RAW264.7 cells at concentrations of 0.1 and 1 µM. Based on these results, all the following experiments were performed at concentrations of 0.1 and 1 µM that did not induce cell toxicity. We next examined the effect of CF on LPS-induced proinflammatory cytokine expressions, such as IL-1β and IL-6, using quantitative real-time polymerase chain reaction (qRT-PCR) analysis (Figure 1C,D). As shown in Figure 1C,D, LPS-induced IL-1β and IL-6 mRNA levels were significantly inhibited by CF pretreatment in a dose-dependent manner. Consistent with these results, CF caused a dose-dependent inhibition of LPS-induced IL-1β and COX-2 protein expression (Figure 1E,F). Taken together, our findings suggest that CF has an inhibitory effect on inflammatory signaling pathways.

### 3.2. CF Does Not Inhibit NF-κB Signaling Pathways

**Figure 2:**
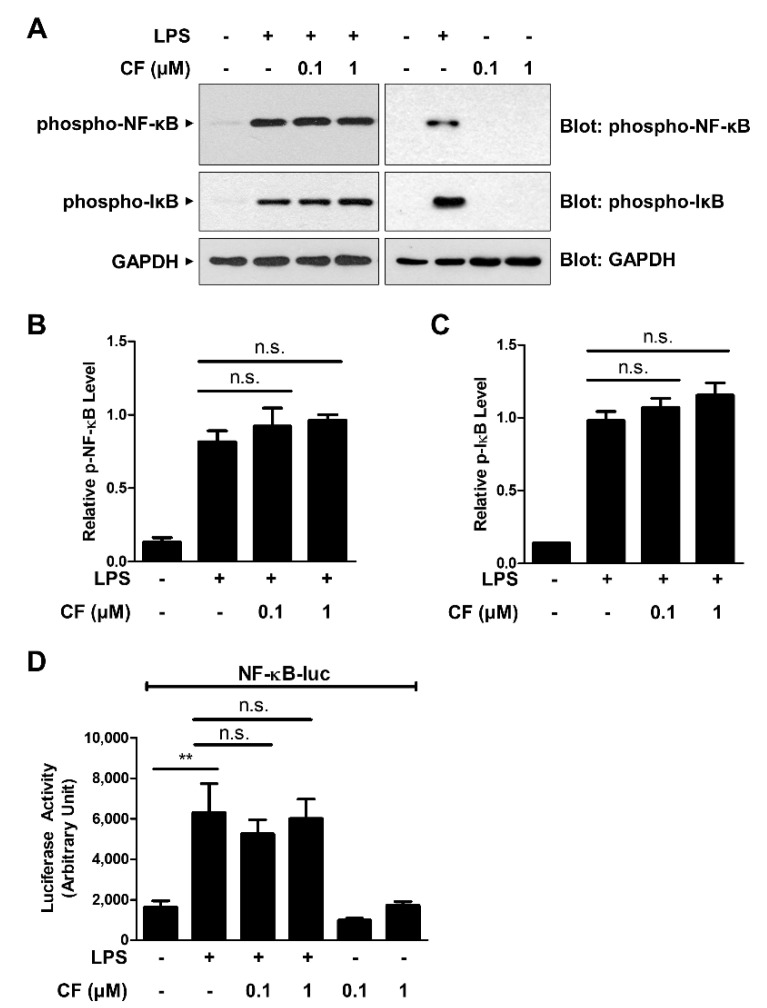
CF does not regulate the NF-κB signaling pathway. (**A**–**C**) RAW264.7 macrophage cells were treated with CF (0.1 and 1 µM) for 30 min before treatment with LPS (500 ng/mL) for 6 h. Western blot analysis was performed with anti-phospho-NF-κB (p65) and anti-phospho-IκB antibodies (**A**) and then quantified (**B**,**C**). (**D**) The NF-κB-luciferase reporter vector was transfected into RAW264.7 cells. After 24 h, the cells were pretreated with CF for 30 min before LPS (500 ng/mL) treatment for 6 h, and luciferase activity was measured. The graphs are the mean ± SD of three separate experiments. ** *p* < 0.01; n.s., nonsignificant.

NF-κB is a key regulator of the expression of numerous inflammatory mediators, such as IL-1β, IL-6, and COX-2. Upon activation, NF-κB is translocated from the cytoplasm to the nucleus, where it binds to specific DNA nucleotide sequences to initiate the transcription of its downstream targets [22]. To determine whether CF exerts an anti-inflammatory effect through the inhibition of NF-κB signaling, we measured the phosphorylation level of NF-κB (p65) by Western blot analysis. As shown in Figure 2A,B, LPS-induced NF-κB phosphorylation was not affected by CF treatment. The phosphorylation of IκB, which releases NF-κB to the nucleus, was consistently unchanged by CF treatment (Figure 2A,C). In addition, NF-κB reporter analysis showed that CF exhibited no relevant changes in LPS-induced NF-κB gene transcriptional activation, indicating that the anti-inflammatory effects of CF were not related to the suppression of NF-κB signaling pathways (Figure 2D).

### 3.3. CF Inhibits the MAPK/AP-1 Signaling Pathway

**Figure 3:**
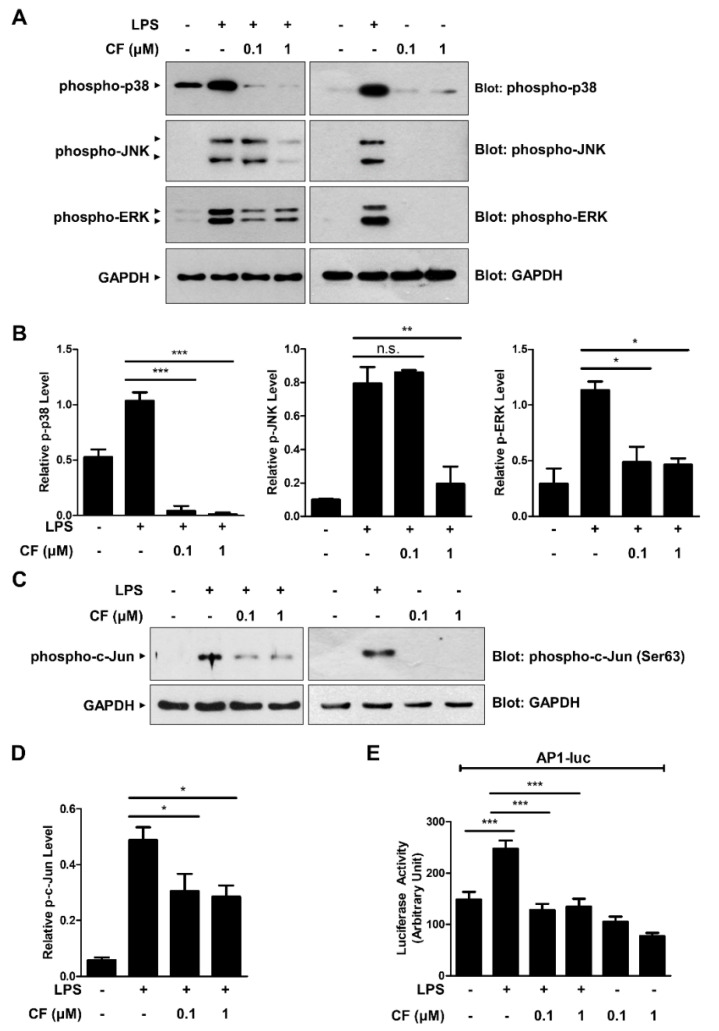
CF regulates the MAPK/AP-1 signaling pathway. (**A**–**D**) RAW264.7 macrophage cells were treated with CF (0.1 and 1 µM) for 30 min before treatment with LPS (500 ng/mL). After 6 h, the cell lysates were subjected to immunoblotting with the indicated antibodies (**A**,**C**) and then quantified (**B**,**D**). (**E**) RAW264.7 macrophages were transfected with the AP-1-luc reporter. After 24 h, the cells were pretreated with CF (0.1 and 1 µM) for 30 min before LPS (500 ng/mL) treatment for 6 h, and luciferase activities in the cell lysates were measured. The results are plotted as the mean ± SD of three individual experiments. * *p* < 0.05; ** *p* < 0.01; *** *p* < 0.001; n.s., nonsignificant.

It is known that MAPKs such as p38, JNK, and ERK play important roles in modulating proinflammatory gene expression. To determine whether CF exerts an anti-inflammatory effect by modulating MAPK signaling pathways, the phosphorylation levels of p38, JNK, and ERK were investigated by Western blot analysis (Figure 3A,B). As shown in Figure 3A,B, significant decreases in the phosphorylation levels of these MAPKs by CF treatment were observed in LPS-triggered RAW264.7 macrophages. Moreover, the phosphorylation of c-Jun, a downstream target of MAPKs, was consistently inhibited by CF treatment (Figure 3C,D). The MAPK activation induces proinflammatory cytokine gene expression by activating the AP-1 transcription factor. We next examined whether the inhibitory effect of CF on MAPK activation was associated with AP-1 transcriptional activation (Figure 3E). AP-1-luciferase reporter analysis revealed that CF effectively suppressed LPS-induced AP-1 transcriptional activation (Figure 3E). These findings suggest that CF exerts an anti-inflammatory effect by modulating the MAPK/AP-1 signaling axis.

### 3.4. CF Suppresses Skin Inflammation in an AD Model

**Figure 4:**
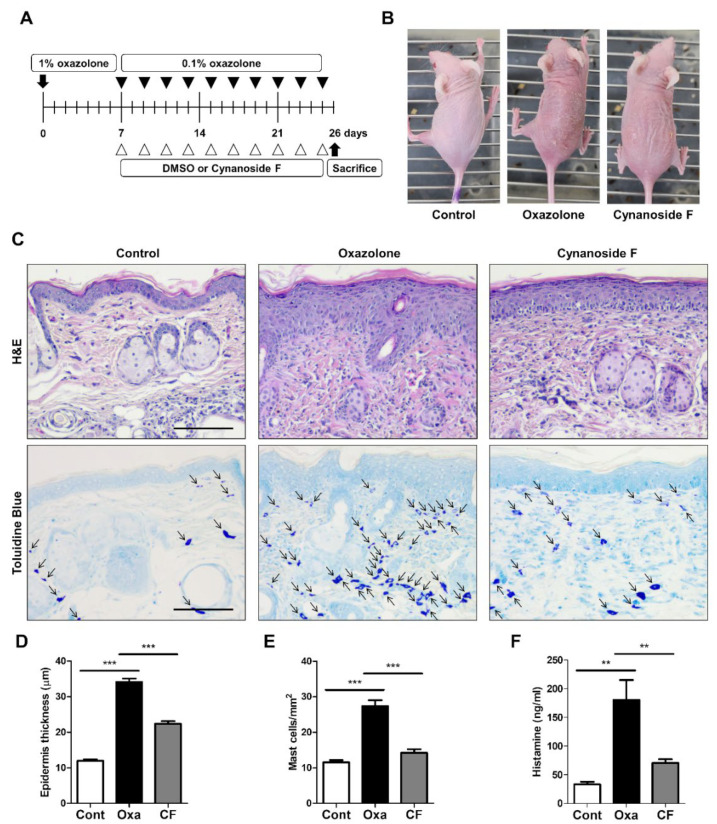
CF inhibits skin inflammation in AD model mice. (**A**) Schematic representation of the experiment. Hairless mice (*n* = 5 per group) were treated with oxazolone together with either vehicle (DMSO) or CF (10 µg/mL). Mice treated with a mixture of acetone and olive oil were used as a control. (**B**) The image shows the dorsal skin of mice on Day 24. (**C**) H&E staining of the epidermis and toluidine blue staining for mast cell infiltration (magnification ×100, scale bar: 50 μm). (**D**) Measurement of the epidermal thickness from five randomized fields. (**E**) Number of mast cells from five randomized fields of each group. (**F**) Histamine levels measured by ELISA. Values are the mean ± SD from five fields in each group (*n* = 5). ** *p* < 0.01; *** *p* < 0.001.

To evaluate the effect of CF on skin inflammation, we used an oxazolone-induced AD murine model. The SKH1 hairless mouse is widely used for various dermatological studies because it can perform experiments without shaving and can easily visualize skin reactions [23]. To ensure that there is no difference in the immunological response according to gender difference, female SKH1 hairless mice were uniformly used. A schematic representation of the experiment using SKH1 hairless mice is depicted in Figure 4A. As shown in Figure 4B, the oxazolone-challenged group showed typical phenotypic symptoms of AD, such as dryness, scaling, and excoriation; however, such skin abrasions were attenuated by the CF treatment (Figure 4B). Consistent with the phenotypic observation, H&E staining revealed that the increased thickness of the epidermis in response to oxazolone, a marker for edema, was significantly reduced in the CF-treated AD mice (Figure 4C,D). We next monitored the infiltration of mast cells into the dermis, where histamine is secreted, by toluidine blue staining (Figure 4C,E). The number of infiltrated mast cells in the dermis of the CF-treated group was significantly decreased compared to that in the oxazolone-treated group (Figure 4C,E). We also measured the histamine level in serum using ELISA (Figure 4F). As shown in Figure 4F, the histamine level was consistently lowered in CF-treated AD mice. These results suggest that CF attenuates skin inflammation in oxazolone-induced AD mice.

### 3.5. CF Suppresses Proinflammatory Cytokine Expression via AP-1 Inhibition in AD Mouse Tissue

**Figure 5:**
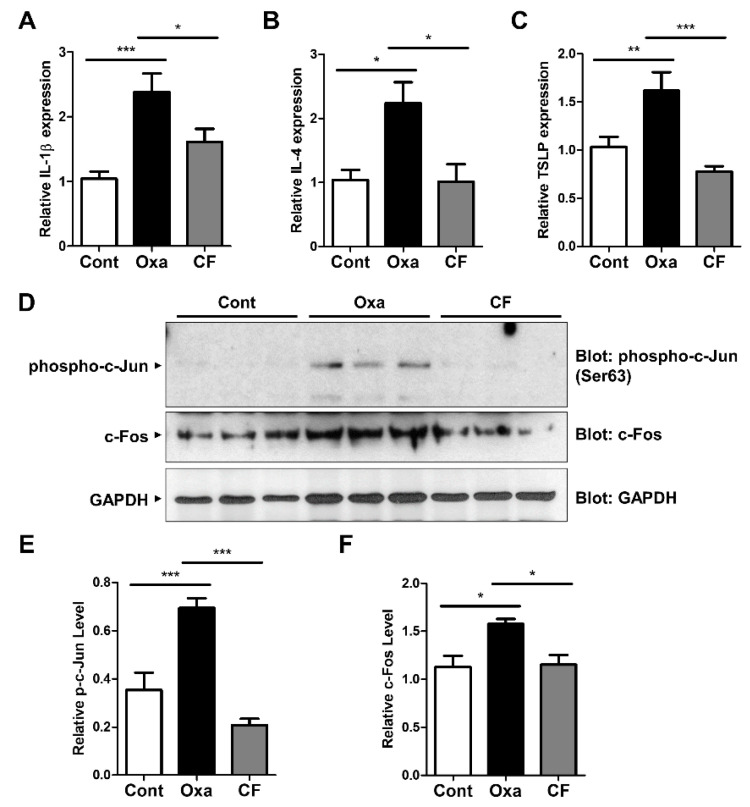
The immunosuppressive effect of CF on AD skin tissues. (**A**–**C**) IL-1β, IL-4, and TSLP mRNA expression levels in skin tissues were measured by qRT-PCR. (**D**–**F**) Western blot analysis was performed with three mice from each group with the indicated antibodies (**D**), and then quantified (**E**,**F**). * *p* < 0.05; ** *p* < 0.01; *** *p* < 0.001.

**Figure 6:**
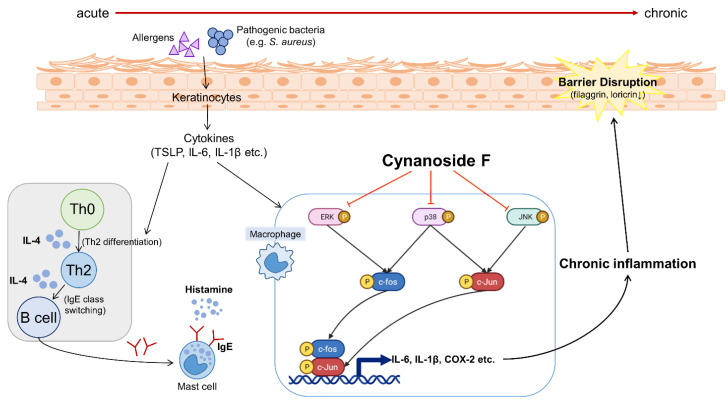
Schematic diagram of the effect of CF on the immunosuppression in AD. CF inhibits the MAPK/AP-1 signaling pathway, which in turn suppresses the expression of proinflammatory mediators in AD skin.

To determine the anti-inflammatory effect of CF in AD mouse tissue, the mRNA levels of proinflammatory cytokines in the skin were measured using qRT-PCR analysis (Figure 5A–C). IL-1β and IL-4 mRNA levels were upregulated in oxazolone-treated AD mouse tissues, and CF application decreased their production (Figure 5A,B). The mRNA level of TSLP, a cytokine that is triggered in many AD-like diseases, was consistently decreased in the group treated with CF (Figure 5C), indicating that CF suppresses proinflammatory cytokine expression in AD tissues. We next examined the c-Jun activation in AD skin (Figure 5D,E). Consistent with the in vitro results, we observed that the phosphorylation of c-Jun was enhanced in oxazolone-treated AD mice and that the level was significantly lowered in CF-treated AD mice (Figure 5D,E). Moreover, we observed that the protein expression of c-Fos, an AP-1 constituent, was consistently decreased in CF-treated AD skin tissues (Figure 5D,F). Collectively, these results indicate that CF ameliorates AD symptoms by suppressing MAPK/AP-1 activation (Figure 6).

## 4. Discussion

AD, a chronic skin disorder with an aggravated immune response, has been on the rise in recent years [24]. T-cell development into Th2 cells results in the production of a significant number of inflammatory cytokines, while mast cells overproduce histamine, resulting in intense pruritus. Excessive scratching destroys the skin barrier, making it vulnerable to skin infections and triggering an exacerbated macrophage-led inflammatory response, which eventually leads to AD [25,26]. Among the diverse pharmacological treatments currently used in AD are topical corticosteroids (TCSs), antihistamines, topical calcineurin inhibitors (TCIs), antibiotics, phototherapy, and systemic immunosuppression. However, effectiveness is frequently proportional to adverse reactions. TCSs, for instance, have been linked to rosacea, skin atrophy, acne, striae, telangiectasias, hyperglycemia, glaucoma, and hypertension [27,28]. Therefore, a search for novel therapeutic strategies with less harmful reactions is necessary.

CF is a pregnane-type compound isolated as a bioactive component from the traditional medicinal plant *Cynanchum atratum*, which belongs to the Asclepiadaceae family, and the root has historically been employed to relieve inflammatory symptoms [29]. At present, more than 400 compounds have been isolated from diverse members of the Asclepiadaceae family, including steroids, acetophenones, saponins, alkaloids, flavonoids, terpenes, polysaccharides, and other compounds [18,19,30]. Pharmacological research has revealed that *Cynanchum atratum*, including its isolated components, possesses anti-inflammatory, cytotoxic, antitumor, and even acetylcholinesterase inhibitory properties [14,17]. Although *Cynanchum atratum* has been shown to have immunosuppressive effects, it is uncertain whether CF has comparable effects and what its intrinsic molecular mechanisms are. In this study, we first report the effect of CF as an immunosuppressor in LPS-induced macrophages and in oxazolone-induced AD mice via inhibition of MAPK signaling and AP-1 transcriptional activity.

When murine macrophages (RAW264.7) were treated with LPS, the expression levels of downstream immune target proteins, such as IL-1β, IL-6, and COX-2, were upregulated, whereas CF treatment inhibited their expression. NF-κB has long been considered a significant transcription factor for controlling the expression of genes involved in inflammatory processes [31]. However, we did not observe an inhibition of the LPS-triggered phosphorylation of proteins such as IκB and NF-κB by CF treatment. Numerous studies have demonstrated the importance of MAPKs in the transcriptional regulation of inflammatory intermediaries via AP-1 transcription factor activation [4,8,32]. We found that CF markedly suppressed LPS-induced phosphorylation of MAPKs, such as p38, JNK, and ERK, and subsequent AP-1 transcriptional activation in a dose-dependent manner. In our previous study, crude extracts of *Cynanchum atratum* and one of the isolated compounds, sinapic acid, exerted an anti-inflammatory function by inhibiting NF-κB signaling [17]. In addition to this report, our current study suggests that CF suppresses LPS-induced inflammatory signaling by inhibiting the MAPK/AP-1 signaling axis. Based on these results, we speculate that the anti-inflammatory activity of *Cynanchum atratum* occurs by regulating multiple signaling pathways, such as NF-κB and AP-1.

To determine the physiological effect of CF, we used a murine AD model. Inflammation was induced by oxazolone, and the oxazolone-treated group showed noticeable symptoms associated with AD. Histologically, there were increases in epidermal thickness, mast cell infiltration, and secreted histamine levels. However, treatment with CF alleviated these symptoms, as seen in other studies where mitigation of AD-like symptoms was observed [4,32]. Furthermore, CF lowered the expression of the inflammatory cytokines such as IL-1β, IL-4, and TSLP in skin tissues. CF consistently inhibited the activation of AP-1, such as c-Jun phosphorylation and c-Fos expression in the AD skin tissues, confirming the in vivo effect of CF.

A wide variety of diseases, including infections, cancer, asthma, gastrointestinal disorders, and even neurological disorders, have been treated with the help of natural extracts [33,34]. According to the previous studies, flavonoids, terpenes, alkaloids, glycosides, and other substances, have been proven safe and effective in the treatment of AD, and a range of them can correct the detrimental alterations of AD [35]. In accordance with these studies, CF could be considered a treatment option for AD. Although our study is the first step to suggest scientific clues for the naturally isolated compound CF, which can suppress inflammatory responses in skin, we believe that our current study is valuable, as it provides fundamental insight into the medicinal value of CF.

## 5. Conclusions

In the present study, we demonstrated that CF attenuates inflammatory signaling by inhibiting MAPK/AP-1 signaling in macrophages and in AD model mice. The finding of the role of CF in regulating inflammatory signaling might facilitate the development of effective treatments for relieving AD pathogenesis. Based on these results, further research will be relevant in determining whether CF is comparable to the well-known standard medicines for the amelioration of inflammatory symptoms in AD patients.

## Figures and Tables

**Table 1:** Sequences of qRT-PCR Primers.

Gene	Primer Sequence	Product Size (bp)
IL-1β	Forward: 5′-CCTCACAAGCAGAGCACAAG-3′ Reverse: 5′-TGTCTTGGCCGAGGACTAAG-3′	203
IL-6	Forward: 5′-TACCACTTCACAAGTCGGAGGC-3′ Reverse: 5′-CTGCAAGTGCATCATCGTTGTTC-3′	116
IL-4	Forward: 5′-ATCATCGGCATTTTGAACGAGGTC-3′ Reverse: 5′-ACCTTGGAAGCCCTACAGACGA-3′	125
TSLP	Forward: 5′-CCCTTCACTCCCCGACAAAA-3′ Reverse: 5′-GCAGTGGTCATTGAGGGCTT-3′	61
β-actin	Forward: 5′-AGAGGGAAATCGTGCGTGAC-3′ Reverse: 5′-CGATAGTGATGACCTGACCGT-3′	138

## Data Availability

Not applicable.
